# Time Outdoors in Nature to Improve Staff Well-Being: Examining Changes in Behaviors and Motivations Among University Staff in the Use of Natural Outdoor Environments Since the Emergence of the COVID-19 Pandemic

**DOI:** 10.3389/fpsyg.2022.869122

**Published:** 2022-07-22

**Authors:** Janet Loebach, Donald A. Rakow, Genevieve Meredith, Mardelle McCuskey Shepley

**Affiliations:** ^1^Department of Human Centered Design, College of Human Ecology, Cornell University, Ithaca, NY, United States; ^2^Section of Horticulture, School of Integrative Plant Science, Cornell University, Ithaca, NY, United States; ^3^Master of Public Health Program, Cornell University, Ithaca, NY, United States

**Keywords:** nature, time outdoors, well-being, university staff, natural outdoor environments, COVID-19, restoration, stress

## Abstract

**Background:**

Work-related stress is of growing concern to employers because of its significant implications for employee dissatisfaction, reduced productivity, and lowered emotional and physical health. Job-related stress is particularly acute among staff members in higher education, negatively impacting the professional work and personal welfare of staff. During the COVID-19 pandemic, stress levels increased, due to work- and non-work-related factors. Work expectations and environments shifted, as did new non-work responsibilities, such as care of dependents. As a result, many people were forced to spend much more time at home. Given the anticipated levels of stress (higher) and the change in time spent at home (increased), we sought to explore if adults were spending more time outdoors, as compared to pre-pandemic times, and if so, for what purposes. We hypothesized that people would be spending more time outdoors in nature during the pandemic, and that they would be doing so to achieve some of the well-documented benefits including managing stress, and bolstering mental health and wellbeing. We further hypothesized that some staff would experience barriers to spending time outdoors in natural outdoor environments (NOEs), potentially limiting their ability to experience these beneficial effects.

**Materials and Methods:**

This study surveyed 507 staff from a large United States university to examine the degree to which staff were spending time in natural outdoor environments (NOEs) during the pandemic (two time-points, compared to pre-pandemic), and whether and how nature-based routines changed as a result of its emergence. The study also examined whether staff were motivated to spend time in nature to improve their mental health and/or wellbeing.

**Results:**

The majority of respondents reported spending more time in NOEs since COVID-19 emerged, particularly early in the pandemic. Respondents reported doing so for restorative purposes, including stress relief, improved mental health, and improved physical health. Relative accessibility of NOEs, both in terms of proximity and number of barriers to access, significantly impacted both time outdoors and the number of NOEs used. Access to safe, high-quality NOEs was not experienced equally across staff respondents; barriers to access tended to be higher among staff living with dependents or others in their household, and for staff who identify as non-White.

**Conclusion:**

Spending time outdoors may have served as a protective factor for many university staff against some of the potentially detrimental effects of the pandemic, particularly reduced mental health and well-being. Universities can contribute to the ongoing well-being of their staff by supporting access to safe, high-quality NOEs on or adjacent to campus. This may also serve to reduce disparities in access to nature and experience of its benefits. Universities may also consider alternative work arrangements for staff to allow for more time for health and wellness self-care during the work day, including spending time outdoors in nature.

## Introduction

In early 2020, the world began to hear of a rapidly spreading virus. Within weeks, SARS-CoV-2 was identified as the highly contagious virus spread by airborne droplets causing COVID-19. Faced with a highly virulent and novel disease that placed a major strain on health systems, public health leaders took unprecedented actions to mitigate spread and keep populations healthy, including limiting numbers of people gathering together and lockdown scenarios.

Emergency public health measures affected many environments, including workplaces. For example, in mid-March 2020, all schools in New York State (NYS) were closed, and all non-essential workers were required to work from home (Johns Hopkins University and Medicine, 2021). In a moment, people’s lives drastically and dramatically changed. School-aged children could no longer go to school, requiring at-home or alternative care arrangements. Workers, if deemed non-essential, needed to find ways to work from home, while also helping dependents to stay engaged in “on-line” learning. Those deemed essential needed to be at work, despite also being tasked with caring for dependents at home; a substantial burden and conundrum. Roughly one-third of employed individuals in the United States worked remotely during the pandemic (U.S. Bureau of Labor Statistics, 2021). The percentage of the population engaged in remote work varied widely across sociodemographic groups: those from households with higher income levels were more likely to work remotely, as were those with more education and in better health (Marshall et al., 2021). Those with lower-wage jobs had less freedom, greater health risks, and more pressures.

### Stress and Work

The workplace has long been viewed as a stressful environment, with worker stress most often associated with an inability to deal adequately with the demands placed upon the individual (Bhui et al., 2016). The novelty and uncertainty of COVID-19, and the related changes in workplace schedules and environments, exacerbated already high levels of stress for many workers. A survey conducted in March, 2020 that found that 88% of workers reported experiencing moderate to extreme stress over the previous 4–6 weeks (Gavidia, 2021). Among those reporting stress, 62% noted losing at least 1 h per day in productivity and 32% lost at least 2 h per day due to COVID-19-related stress. While lockdowns were necessary to mitigate the spread of COVID-19 (Sault, 2020) and to reduce pressure on health care systems, other research suggested serious secondary impacts as people continued to be confined to their homes (Evanoff et al., 2020). In response to remote work, one study found that those employees performing work deemed “non-essential” during the crisis phase of the pandemic experienced a high prevalence of stress, anxiety, depression, work exhaustion, burnout, and worsened well-being approximately 4–5 weeks after work-from-home policies were implemented (Evanoff et al., 2020).

### Effects of Stress

Stress, caused by day-to-day activities, or traumatic events, has serious impacts on personal and public health and wellbeing. The COVID-19 pandemic has been identified as a traumatic event (Centers for Disease Control and Prevention, 2021). Whether infected with SARS-CoV-2 or not, people may have experienced increased feelings of uncertainty, anxiety, irritation, anger, or denial. In the short-term, people may feel tired, overwhelmed, burned out, sad, and even depressed; may lack motivation or experience insomnia (Marelli et al., 2021); and may display an inability to concentrate (Centers for Disease Control and Prevention, 2021). Eighteen months after the onset of the pandemic, 32% of surveyed adults indicated that they are sometimes so stressed by COVID-19 that they struggled to make basic decisions. Stress levels were especially high among parents with children under age 18, 47% of whom stated that both day-to-day and major decision making was more stressful than it was pre-pandemic (American Psychological Association, 2021). If left unaddressed, experiencing such stressors can lead people to engage in unhealthy behaviors, such as increased use of alcohol, tobacco, or other drugs (Centers for Disease Control and Prevention, 2021). Elevated stress is also linked to chronic diseases such as heart disease and stroke (Science Daily, 2017). People with high levels of stress may struggle to attend to personal and family needs while working; managing a different workload and/or lacking access to tools and equipment needed to perform work can further exacerbate negative feelings (Nigam et al., 2020).

### Managing Stress by Spending Time Outdoors in Nature

Presently, many Americans do not have strong stress management skills or coping mechanisms. The American Psychological Association’s (2021) “Stress in America” report found that 45% of young adults in their twenties and 50% of millennials said they do not know how to manage their stress due to the coronavirus pandemic (American Psychological Association, 2021). Adults were even less likely to feel that they are doing enough to manage their stress or to feel that their mental health was very good or excellent. In particular, Hispanic and Black adults were less likely to say they are faring well during the coronavirus pandemic than non-Hispanic White adults (American Psychological Association, 2021).

Considerable evidence has demonstrated that spending time outdoors in nature can be an effective way of managing stress and bolstering mental health. Humans derive both psychological and physiological benefits from accessing nature, including reductions in overall stress levels (Fan et al., 2008; Antonelli et al., 2019; Hunter et al., 2019); anxiety (Bratman et al., 2015); rumination (Bratman et al., 2012); and depression (Frumkin et al., 2017; Kondo et al., 2018).

However, despite the myriad benefits of time outdoors and in nature, not all populations have easy access to natural outdoor environments either at or near their home. Certain populations, including older adults and racial and ethnic minorities, lack the ability to easily access natural environments at or near home, or feel safe and welcomed in these settings (Rowland-Shea et al., 2020; Lockhart et al., 2021). Young people of color face a complex matrix of external, socioeconomic, and psychological barriers that limit their engagement with nature. Barriers to access involve factors such as work schedules or home responsibilities that preclude them from carving out time to spend in nature, or lack of access to natural outdoor environments with amenities that support their interests and are clean and well-maintained (Ibes et al., 2021). If time in nature is to be considered as a tool to support mental health and wellbeing in a systematic and equitable way, understanding barriers to access is key, as access is associated with the quality or frequency of outdoor experiences (Ibes et al., 2021).

### University Staff Managing During COVID-19

There is mounting evidence that job-related stress is particularly acute among staff members of colleges and universities. University employees, including general and academic staff, report that work is a significant cause of stress in their lives (Gillespie et al., 2001; Kinman and Wray, 2013). University staff indicate that their level of stress is high or very high, and many experience levels of stress they find unacceptable (Kinman and Wray, 2013). Further, university employees report that job-related stress has had a deleterious impact on their professional work and personal welfare (Gillespie et al., 2001; Hogan et al., 2002). During COVID-19, university staff reported emergent challenges due to COVID-related social isolation: lack of personal interactions, lack of motivation, and anxiety, boredom and loneliness (Leal Filho et al., 2021). Even as COVID-19 restrictions are lifted, some university staff may continue to work remotely (Ellis, 2021).

As COVID-19 emerged, university staff in NYS were classified into essential or non-essential groups, and the work environment changed dramatically. This already highly stressed population faced new and variable stressors, depending on their living context. We anticipated that this drastic change would influence people differently, that new work expectations or locations might influence actions, and that different or new coping mechanisms may have been utilized to manage stress or uncertainty. In short, we hypothesized that during the pandemic, some people would be spending more time outdoors (as compared to pre-pandemic), and that they would be doing so to achieve some of the well-documented benefits: managing stress and bolstering mental health and wellbeing. Specifically, we sought to explore the degree to which staff were spending time in natural outdoor environments (NOEs) during the pandemic, and for what purpose. We also sought to determine whether and how their nature-based routines changed as a result of the emergence of COVID-19. We further hypothesized that some staff would experience barriers to spending time in NOEs, potentially limiting their ability to experience these beneficial effects.

To test our hypotheses, we engaged staff at a large university in New York state NYS. We specifically sought to explore (1) how much time staff spent outdoors during COVID-19 (as measured in Fall 2020), and if that differed from routines pre-COVID-19 (Fall 2019) or soon after COVID-19 emerged (Spring 2020); (2) what types of NOEs were used by staff during the pandemic vs. pre-COVID-19; (3) what types of outdoor activities staff engaged in and how that changed during COVID-19; and (4) motivations for spending time outdoors before and since the pandemic emerged. To better understand variations in or barriers to time outdoors, we explored differences in staff access to various types of NOEs, as well as in their home setting, work location, and race.

By understanding whether time outdoors in nature has been an intentional and valuable strategy for improved mental health during the pandemic, we hope to be able to raise awareness among employers, such as universities, about the benefits of facilitating time outdoors in nature among their employees, and the barriers which may need to be addressed to do so.

## Materials and Methods

Data for this study were collected via an anonymous online survey distributed to staff in a large research university in NYS (student population ∼ 25,000) in NYS. Between March 2020 and July 2020 most staff were expected to work remotely from home. The time leading up to the study period (Fall 2020) remained a time of uncertainty related to COVID-19: vaccinations were not yet available, and the COVID-19-related mortality rate in the United States remained high. As the study was administered (November and December 2020), NYS had experienced close to one million cases, and COVID-19 rates were increasing (New York State, 2020). At the university where the study took place, a hybrid education model was in use which offered both online and in-person classes; only about 75% of students opted for in-person classes in Fall 2020. However, at this time an estimated 60% of staff were still working remotely from home.

The survey contained 28 questions related to access to NOEs, time spent outdoors in nature, types of outdoor activities in which staff engaged, reasons for spending time in nature, and perceived benefits of time in nature. For most questions respondents were asked to report on experiences and actions related to three time points: recent experience/mid-COVID (Fall 2020), pre-COVID (Fall 2019), and early-COVID (April-July 2020) or else activities and perceptions “before COVID-19 emerged” or “since COVID-19 emerged.” Staff were also asked to speak to outdoor access and routines both from home and while on campus; however, for the purpose of this paper only home-based routines were included for analysis. To stratify responses, staff answered 9 additional questions related to demographics, employment status, and home location. Survey questions were developed from other available tools, reviewed by context and evaluation experts, and then beta tested for validity. The final survey (Supplementary Appendix 1) comprised mostly multiple choice (single answer) and multiple response (check all that apply) questions, complemented by three open-ended short-answer questions; no questions were forced. After being vetted by Human Resources administrators, the survey was programmed into Qualtrics. As programmed, the survey took respondents approximately 10–15 min to complete.

The survey was open and accessible to staff from November 25 through December 31, 2020. An invitation and link to participate in the survey was distributed via an online “wellness” newsletter with a distribution list of ∼7,000 staff members employed at the university’s primary campus. The survey invitation indicated that all participants would be entered in a drawing for one of 35 gift cards (value $50). A total of 507 valid surveys were collected from staff members.

Means and standard deviations are reported for continuous data, percentages are presented for categorical data (R Core Team, 2021). Associations between two categorical variables were assessed using Fisher’s Exact Test. *T*-tests and ANOVAs were used to compare continuous measurements across groups. Paired *t*-tests were used to assess the within-subject change of continuous variables whereas McNemar’s test was used to assess the within-subject change of binary variables over the two time frames (before and since the emergence of COVID-19). Generalized linear mixed models with a binary distribution with a random effect of subject and a fixed effect of time were used to assess the overall difference of participation in outdoor activities and use of natural outdoor environments (NOEs) over the two time points. No control variables were added to regression models. Pearson correlations were used to assess the relationship between continuous variables, whereas point-biserial correlations are reported for relationships between a continuous and binary variable. Data were analyzed using the complete variable data for individual hypotheses; no imputation was carried out to account for missing data (individual response rates are indicated in each table as appropriate). All analyses were completed using R (4.1.1 Kick Things; 2021).

## Results

### Participants

A total of 507 valid surveys were collected from staff members (see Table 1). At the time of data collection in December 2020, 50% of respondents were still working fully remotely from home, 38% were splitting their time in some way between home and campus, and only 11% were working fully from campus (see Table 1). The participant sample was largely comprised of staff who identify as female (82%, compared to 57% of all university staff) and White (78%, compared to 85% of all staff). Just over half (54%) of participants were salaried employees, and 43% were hourly employees. Roughly one-third of participants came from each urban (32%), suburban/small town (41%), and rural home community settings (27%). The vast majority (95%) were living with others in their household at the time of the study.

**TABLE 1:** Participant demographics.

	Proportion of participants (%) *n* = 507	Proportion of all staff (%)* *n* = 7420*
**Gender**		
*Female*	82	57
*Male*	16	43
*Other*	0.4	Unknown
*Prefer not to answer*	1.1	
**Race**		
*White*	78	85
*Non-White*	22	15
**Work location**		
*Remote*	50	60
*Hybrid*	38	Unknown
*On campus*	11	Unknown
*Other*	1	Unknown
**Position category**		
*Salaried*	54	48
*Hourly*	43	52
*Other*	3	Unknown
**Home setting**		
*Urban*	32	Unknown
*Suburban/Small town*	41	Unknown
*Rural*	27	Unknown
**Living arrangements**		
*Living alone*	5	Unknown
*Living with others*	95	Unknown

*At the time of the study, Fall 2020.

### Time Spent Outdoors in Nature

On average, respondents spent substantial time outdoors during the Fall of 2020; 88% reported spending time outdoors on two or more days per week on average, and 62% typically spent time outdoors on four or more days per week (see Table 2). Over 80% of respondents spent at least 30 min outdoors each day they were out; 98% reported spending at least 15 min outdoors on each day they spent time outdoors. While these proportions are high, almost half (47%) of respondents indicated that they spent even more time outdoors in the early days of the pandemic in Spring 2020 when the campus was largely closed and most respondents were working remotely. Reasons for this difference given by respondents included: the better weather conditions and increased daylight in the Spring compared to late Fall; the increased free time in their schedule because of reduced social activities (consequences of early pandemic lockdowns) and commuting time, and in some cases, lighter workloads; the need to get outdoors for exercise; and having a mental reset or a safe way to socialize with others. However, a portion of respondents (16%) spent less time outdoors early in the pandemic, citing ongoing fears associated with limited understanding of COVID-19 transmission, the lack of companions to spend time with outdoors, and limited access to nature.

**TABLE 2:** Time spent outdoors in nature.

Time outdoors/In nature		Home setting				Work location					Living arrangements			Race		
	All *n* = 507 (%)	Rural *n* = 121 (%)	Suburb/Small town *n* = 184 (%)	Urban *n* = 143 (%)	p+	Remote *N* = 246 (%)	Hybrid *N* = 184 (%)	On campus *N* = 52 (%)	Other *N* = 6 (%)	p+	Live alone *N* = 24 (%)	Live with others *N* = 483 (%)	*p*	Non-white *N* = 113 (%)	White *N* = 394 (%)	*p*
***Average time outdoors per week Fall 2020***					0.014*					0.6			0.8			0.13
*No days*	1	0	0.5	2.1		0	2.2	1.9	0		0	1		1.9	0.8	
*1 day*	11	5	10	15		9.8	9.8	13	17		16	10		16	9.1	
*2–3 days*	27	30	28	26		27	26	27	33		32	27		26	27	
*4–6 days*	35	28	40	34		37	36	25	17		21	36		37	35	
*7 days*	27	35	21	24		26	26	33	33		32	27		19	29	
*Staff spending 4+ days outdoors per week*	62	65	61	57	0.4	63	62	58	50	0.9	53	62	0.8	56	63	0.2
***Average time spent outdoors per outing Fall 2020***	*n* = 494				0.014*					0.6			0.8			0.2
*Less than 15 min*	2	1.7	1.6	2.9		2.8	1.7	0	0		0	2.1		1.9	2	
*15–30 min*	17	12	13	26		17	14	16	50		25	17		23	15	
*31 min – 1 h*	48	45	55	45		48	50	41	17		44	48		40	50	
*More than 1 h*	34	41	30	26		32	34	43	33		31	34		35	33	
*Staff spending 30+ min outdoors per outing*	81	87	85	71	0.009**	84	84	50	80	0.6	75	81	0.8	75	83	0.13
***Time outdoors Spring 2020 (vs. Fall 2020)***	*n* = 499									0.9			0.8			0.13
*Less time*	16	1.7	2.7	2.8	0.4	17	15	12	33		6.2	2.5		20	15	
*About the same time*	34	16	13	22		35	33	38	17		31	16		32	34	
*More time*	47	37	33	31		46	49	48	50		19	34		42	49	
*I don’t know*	2.6	45	52	45		2.8	2.7	1.9	0		44	48		5.7	1.8	
***Time outdoors Fall 2019 (vs. Fall 2020)***	*n* = 499				0.009**					0.6			0.8			0.13
*Less time*	40	55	32	36		41	52	33	37		31	40		32	42	
*About the same time*	2.2	1.7	2.2	2.1		1.6	1.9	0	2.8		0	2.3		3.8	1.8	
*More time*	34	31	38	34		30	21	33	40		44	34		32	35	
*I don’t know*	24	13	28	27		27	25	33	20		25	24		31	22	

*+Fisher’s exact test across groups; adjusted p-values.*

p-values: *** p < 0.001; **p < 0.01; *p < 0.05.

While 40% of respondents indicated that they spent more time outdoors in Fall 2020 than same time the previous year before the pandemic emerged (Fall 2019), 34% of respondents spent less time outdoors than in Fall 2019. Respondents who were spending more time outdoors in Fall 2020 cited increased free time due to reduced commuting time or job changes or loss, as well as increased need for and appreciation of being outdoors in nature to support their mental health. Reasons for spending less time outdoors since the pandemic emerged included increased workload since the onset of COVID-19, avoiding crowds or other people due to ongoing fears around the virus, and mental health struggles which made it more difficult for some respondents to leave their homes.

Patterns in time outdoors also varied significantly depending on type of home community or home setting (see Table 2). Respondents living in rural and suburban/small town settings were more likely to spend at least 30 min outdoors each day out than respondents living in more urbanized areas (*p* = 0.014), but there were no significant differences by home setting in the proportion who spent four or more days outdoors per week. Furthermore, time spent outdoors did not vary significantly for respondents working remotely versus those working on campus, or among those living on their own versus with others, or between White and non-White respondents.

There were no significant differences across any of the subgrouping examined for the time spent outdoors Fall 2020 versus Spring 2020 (see Table 2), suggesting similar changes in use across subgroups since the pandemic emerged. However, significantly more rural dwelling respondents (55%) indicated their time outdoors had increased when comparing Fall 2020 to the previous Fall season (2019) than either suburban/small town (32%) or urban (36%) colleagues (*p* = 0.009).

### Changes in Outdoor Activities

The most common outdoor activities both before and since the pandemic emerged were lower intensity activities (91%) such as walking, gardening, birdwatching or fishing, and these levels were stable over time both between and within participants (see Table 3). Approximately half of participants engaged in higher intensity activities, such as running, biking, rock climbing or kayaking, both before (52%) and since (48%) COVID-19 emerged; however, there was a significant decrease in such activities across all participants (*p* = 0.028) since the pandemic began. Not surprisingly due to COVID-19-related restrictions, engagement in social activities (e.g., gathering or dining with others) significantly dropped since COVID-19 emerged (*p* < 0.001). Restorative activities outdoors, however, such as resting, reading, or meditating significantly increased over pre-pandemic levels across all participants (*p* < 0.001). Changes across all participants were mirrored by activity changes within individuals as well. There was a significant increase in restorative activities outdoors (*p* < 0.001) and decreases in both social activities (*p* < 0.001) and higher intensity (*p* = 0.05) since the onset of COVID-19. Overall, there was a significant decrease in the average number of different types of outdoor activities in which respondents engaged (*p* < 0.001) from 2.63 (SD = 1.05) to 2.42 (SD = 1.05).

**TABLE 3:** Changes in types of outdoor activities.

	Across participants				Within participants			
Outdoor activity types	Regularly engaged in before COVID-19 emerged	Regularly engaged in since COVID-19 emerged	Odds ratio (95% CI)	p+	Engaged in before but not since	No change in engagement	Engaged in since but not before	p++
	*n* = 507 (%)	*n* = 507 (%)			*n* = 507 (%)	*n* = 507 (%)	*n* = 507 (%)	
Social (e.g., gatherings or dining with others)	72	44	3.39 (2.58–4.47)	<0.001***	41	47	12	<0.001***
Lower intensity (e.g., walking, hiking, gardening, birdwatching, fishing)	91	91	1 (0.65–1.53)	1.0	4.7	91	4.7	1
Higher intensity (e.g., running, biking, rock-climbing, kayaking)	52	48	1.46 (1.07–1.98)	0.028*	11	81	7.1	0.05*
Restorative (e.g., resting, reading, meditating, sleeping)	45	57	0.36 (0.24–0.53)	<0.001***	4.5	79	16	<0.001***
Other	0.6	0.8	0.15 (0.002–11.4)	0.5	0.2	99	0.4	1
**Average number of types of outdoor activities (SD)**	**2.63 (1.05)**	**2.42 (1.05)**		p+++ < 0.001***				

*+Generalized linear mixed models across participants; adjusted p-values.*

*++McNemar’s chi squared test within participants; adjusted p-values.*

*+++Paired t-test.*

p-values: ***p < 0.001; ** p < 0.01; *p < 0.05.

### Changes in Use of Natural Outdoor Environments

Participants reported regularly using an average of 4.93 (SD = 2.26) different types of NOEs before the pandemic emerged (see Table 4). The most common spaces used pre-pandemic were NOEs at home: private or shared yards (85%) and decks, balconies or patios (76%). The most common public NOEs were nature/hiking trails (71%), rivers, streams, canals or waterfalls (61%), and public parks, gardens or orchards (56%). Across participants, use levels generally remained stable after the pandemic emerged, with no significant changes. Within subject changes mirrored those found across the participant sample. Individual respondents’ use of for each type of NOE also remained quite stable. The average total of different types of NOEs used decreased slightly to 4.65 (SD = 2.29; *p* = 0.04) from pre-COVID-19 levels (4.76; SD = 2.29) but the difference was not significant.

**TABLE 4:** Changes in the use of natural outdoor environments (NOEs).

Type of natural outdoor environments (NOE)	Across participants				Within participants			
	Use before COVID-19 emerged	Use since COVID-19 emerged	Odds ratio (95% CI)	p+	Used before but not since	No change in use	Used since but not before	p++
	*n* = 507 (%)	*n* = 507 (%)			*n* = 507 (%)	*n* = 507 (%)	*n* = 507 (%)	
Private or shared yard	85	83	1.14 (0.82–1.6)	0.63	5.9	90	4.1	0.47
Deck, balcony, or patio	76	75	1.05 (0.79–1.4)	0.90	6.1	89	5.1	0.77
Public park, garden, or orchard	59	56	1.33 (0.94–1.88)	0.22	13	78	9.5	0.25
Botanical garden, arboretum, or nature center	36	31	1.55 (1.08–2.23)	0.10	13	78	8.3	0.09
Nature/Hiking trail	71	71	1 (0.68–1.47)	1	9.9	80	9.9	1
Woodland or conservation area	50	52	0.83 (0.61–1.13)	0.40	7.3	83	9.3	0.5
River, stream, canal, or waterfall	61	57	1.39 (0.94–2.05)	0.22	10	83	6.7	0.24
Lake, pond, or beach	51	47	1.52 (1.12–2.07)	0.08	10	84	5.7	0.09
Other	2.4	2.4	1 (0.45–2.25)	1	0.6	99	0.6	1
**Average number of NOEs used (SD)**	**4.93 (2.26)**	**4.76 (2.29)**		**p+++ = 0.13**				

*+Generalized linear mixed models across participants; adjusted p-values.*

*++McNemar’s chi squared test within participants; adjusted p-values.*

+++Paired t-test.ȷp-values: *** p < 0.001; **p < 0.01; *p < 0.05.

Considering differences in NOE use, patterns again differed across some respondent subgroups (see Table 5). Rural dwellers reported higher usage of private or shared yards than their suburban/small town and urban colleagues both before (*p* < 0.001) and since (*p* = 0.031) COVID-19 emerged, and urban respondents reported significantly higher use of public parks, gardens or orchards as well as rivers, streams, canals or waterfall areas both before (*p* < 0.001) and since (*p* < 0.001) the beginning of the pandemic. The usage of botanic gardens, arboreta and nature centers remained stable among urban respondents, but decreased among both rural and suburban/small town dwellers from pre-COVID-19 levels. The average number of different NOEs used by rural and suburban/small town respondents decreased slightly since COVID-19 emerged, but otherwise there were no significant differences in the average total number of NOEs used before or after COVID-19 emerged based on the type of home setting.

**TABLE 5:** Differences in natural outdoor environment (NOE) use by subgroups.

Type of NOE	Time point	Home setting				Work location					Living arrangements			Race		
		Rural	Suburb/Small town	Urban	p+	Remote	Hybrid	On campus	Other	p+	Live alone	Live with others	p+	Non-White	White	p+
		*n* = 121 (%)	*n* = 184 (%)	*n* = 143 (%)		*n* = 246 (%)	*n* = 184 (%)	*n* = 52 (%)	*n* = 6 (%)		*n* = 24 (%)	*n* = 483 (%)		*n* = 113 (%)	*n* = 394 (%)	
Private or shared yard	Before	97	88	78	<0.001***	87	84	87	100	0.9	58	86	0.022*	72	89	<0.001***
	Since	93	85	78	0.031**	83	85	87	83	>0.9	58	84	0.033*	70	87	<0.001***
Deck, balcony or patio	Before	80	79	71	0.33	76	77	75	67	0.9	58	76	0.167	65	79	0.022*
	Since	76	77	74	0.9	72	79	77	83	0.6	58	75	0.218	65	77	0.051
Public park, garden or orchard	Before	50	54	73	<0.001***	60	60	60	67	>0.9	33	61	0.06	62	59	0.7
	Since	43	49	76	<0.001***	58	56	48	33	0.6	42	57	0.37	56	56	>0.9
Botanical garden, arboretum, or nature center	Before	29	36	40	0.33	33	39	44	50	0.6	29	37	0.61	37	36	0.84
	Since	17	30	41	<0.001***	29	34	33	0	0.6	29	31	>0.9	35	30	0.52
Nature/Hiking tail	Before	71	70	71	>0.9	70	71	79	100	0.6	50	72	0.13	65	72	0.29
	Since	69	75	71	0.67	69	74	71	67	0.88	58	71	0.37	60	74	0.035*
Woodland or conservation area	Before	63	42	48	0.005**	46	55	54	50	0.6	38	51	0.37	44	52	0.29
	Since	61	47	51	0.13	48	60	52	17	0.31	42	53	0.47	43	55	0.103
River, stream, canal, or waterfall	Before	60	54	69	0.07	58	64	63	50	0.8	54	61	0.61	54	62	0.26
	Since	57	51	68	0.036*	55	62	56	33	0.53	50	58	0.61	49	60	0.103
Lake, pond, or beach	Before	54	51	48	0.89	48	53	60	33	0.6	54	51	0.88	50	52	0.84
	Since	53	44	43	0.33	46	51	40	17	0.53	58	47	0.47	44	48	0.61
Other	Before	2.5	1.6	2.8	0.9	2.8	1.1	3.8	17	0.4	4.2	2.3	0.59	4.4	1.8	0.29
	Since	2.5	1.6	2.8	0.9	2	2.2.	5.8	0	0.6	0	2.5	>0.9	3.5	2	0.41
**Average total number of NOEs used (SD)**	**Before**	**5.12 (2.01)**	**4.76 (2.11)**	**5.01 (2.40)**	**0.45++**	**4.82 (2.17)**	**5.06 (2.24)**	**5.27 (2.31)**	**5.33 (1.75)**	**0.71++**	**3.79 (3.05)**	**4.98 (2.20)**	**0.06++**	**4.54 (2.65)**	**5.04 (2.12)**	**0.103++**
	**Since**	**4.77 (2.14)**	**4.61 (2.17)**	**5.05 (2.24)**	**0.33++**	**4.62 (2.28)**	**5.05 (2.14)**	**4.71 (2.31)**	**3.33 (2.16)**	**0.40++**	**3.96 (3.11)**	**4.80 (2.23)**	**0.22++**	**4.26 (2.71)**	**4.90 (2.13)**	0.035*++
	**p+++**	**0.13**	**0.45**	**0.90**		**0.4**	**0.97**	**0.32**	**0.24**		**0.78**	**0.13**		**0.26**	**0.28**	

*+Fisher’s exact test across groups; adjusted p-values.*

*++ANOVA; adjusted p-values; adjusted p-values.*

*+++Paired t-test Before vs. Since; adjusted p-values.*

p-values: ***p < 0.001; **p < 0.01; *p < 0.05.

There were also no significant differences in the use of each type of NOE nor the average number of NOEs used across respondent groups working remotely versus on-campus or some hybrid of the two. However, the average number of different NOEs used by both remote on-campus respondents decreased slightly since the emergence of the pandemic. Only a small proportion of respondents reported living alone at the time of the survey, but those who did used private or shared yards much less than those with others in their household both before (*p* = 0.022) and since the pandemic (*p* = 0.033). Those living alone also generally reported less time on average in each NOE type than their colleagues with others in their households, but the mean number of NOEs used increased slightly since the onset of COVID-19 (3.79 (SD = 3.05) to 3.96 (SD = 3.11), partially closing the gap in use compared to colleagues living with others.

Respondents identifying as White used NOEs at home (e.g., private or shared yards, decks, balconies) significantly more than non-White respondents both before (*p* < 0.001) and since (*p* < 0.001) the pandemic (see Table 5). While both groups showed an overall slight decrease in the average number of NOEs used since the pandemic, each group’s levels of use for each type of NOE remained fairly stable. The exception was that non-White respondents decreased their use of nature/hiking trails since COVID-19 emerged, making their use of this NOE significantly less than White respondents (*p* = 0.035). White respondents used a greater diversity of NOEs on average than non-White respondents both before and since the pandemic, but this gap increased to a significant level (*p* = 0.035) with the onset of COVID-19.

### Changes in Motivations for Spending Time Outdoors

Before COVID-19 emerged, a high proportion of respondents indicated they spent time outdoors for exercise or to improve their physical health (86%), for fun or recreation (85%), and for stress relief or improved mental health (78%) (see Table 6). The average number of different reasons for spending time outdoors did not significantly change once the pandemic emerged, however, there was a significant increase in the overall number of respondents choosing to spend time outdoors to support their mental health (87%; *p* < 0.001). Spending time outdoors to socialize with friends and for fun or recreation also decreased since the pandemic, but these differences were not significant. These trends were also reflected in changes within individuals: more respondents reported choosing to spend time outdoors for improved mental health since the onset of COVID-19, and fewer chose to spend time outdoors for fun or to socialize with friends.

**TABLE 6:** Changes in motivations for spending time outdoors in nature.

Motivations for spending time outdoors	Between subjects change				Within subjects change			
	Motivation before COVID-19 emerged	Motivation since COVID-19 emerged	Odds ratio (95% CI)	p+	Motivation before but not since	No change in motivation	Motivation Since but not before	p++
	*n* = 507 (%)	*n* = 507 (%)			*n* = 507 (%)	*n* = 507 (%)	*n* = 507 (%)	
For exercise/Improved physical health	86	86	0.98 (0.69–1.41)	1	4.7	90	4.9	1
For stress relief/Improved mental health	78	87	0.51 (0.37–0.72)	0.001***	4.1	82	14	<0.001***
For fun/Recreation	85	80	1.36 (0.98–1.88)	0.268	9.5	85	5.1	0.049*
To have contact with nature	74	75	0.94 (0.71–1.25)	1	4.5	90	5.7	0.68
To spend time with family	58	59	0.95 (0.74–1.22)	1	11	76	12	0.76
To socialize with friends	61	55	1.29 (1–1.65)	0.246	23	60	17	0.084
To Do yard/Farm work	1.2	1.2	1 (0.32–3.12)	1	0	100	0	n/a
To garden	0.6	0.6	1 (0.20–4.98)	1	0	100	0	n/a
To care for animals	3.2	3.2	1 (0.50–2.02)	1	0	100	0	n/a
For other work-related activities	1.2	0.6	2.01 (0.5–8.09)	0.975	1	99	0.4	0.68
Other	0.4	0.6	0.67 (0.11–4)	1	0	100	0.2	1
**Mean number of total motivations mean (SD)**	**4.48 (1.60)**	**4.50 (1.65)**		**p+++ = 1** (−0.12−0.14)				

*+Generalized linear mixed models across participants; adjusted p-values.*

*++McNemar’s chi squared test within participants; adjusted p-values.*

*+++Paired t-test.*

p-values: ***p < 0.001; **p < 0.01; *p < 0.05.

### Factors Influencing Time Spent Outdoors and Use of Natural Outdoor Environments

Several factors appeared to impact the amount of time respondents spent outdoors since the emergence of the pandemic as well as respondents use of NOEs. Respondents who indicated they generally experience more positive feelings after spending time outdoors were significantly more likely to spend more time outdoors per week (*p* < 0.001; 95% CI: 0.04–0.22) and for longer durations on each outing (*p* < 0.001; 95% CI: 0.12–0.29). Similarly, positive feelings after time outdoors were correlated with respondents’ use of NOEs both before (*p* < 0.001; 95% CI: 0.07–0.24) and since (*p* < 0.001; 95% CI: 0.03–0.21) COVID-19 emerged (see Table 7).

**TABLE 7:** Factors influencing time spent outdoors and use of NOEs.

Factor	Time spent outdoors Fall 2020		Use of NOEs	
	Spent 4+ days outdoors *r*, (95% CI), p+	Spent 30+ min outdoors per outing *r*, (95% CI), p+	Total NOEs used before COVID-19 emerged *r*, (95% CI), p+	Total NOEs used since COVID-19 emerged *r*, (95% CI), p+
***Feel after total score***	*r* = 0.13 (0.04, 0.22), *p* = 0.004**	*r* = 0.20 (0.12, 0.29), *p* < 0.001***	*r* = 0.16 (0.07, 0.24), *p* < 0.001***	*r* = 0.12 (0.03, 0.21), *p* < 0.001***
Happier	*r* = 0.10 (0.01, 0.19), *p* = 0.024*	*r* = 0.13 (0.05, 0.22), *p* = 0.002**	*r* = 0.15 (0.06, 0.24), *p* < 0.001***	*r* = 0.09 (0.005, 0.18), *p* = 0.039*
Healthier	*r* = 0.09 (0.003,0.18), *p* = 0.042*	*r* = 0.18 (0.09, 0.27), *p* < 0.001***	*r* = 0.15 (0.06, 0.23), *p* = 0.001**	*r* = 0.12 (0.03, 0.21), *p* = 0.007**
Less stressed/Anxious	*r* = 0.12 (0.03, 0.21), *p* = 0.008**	*r* = 0.18 (0.09, 0.26), *p* < 0.001***	*r* = 0.14 (0.05, 0.22), *p* = 0.002**	*r* = 0.09 (0.003, 0.18), *p* = 0.041*
More focused	*r* = 0.15 (0.06, 0.24), *p* = 0.001**	*r* = 0.19 (0.10, 0.27), *p* < 0.001***	*r* = 0.12 (0.03, 0.20), *p* = 0.011*	*r* = 0.12 (0.03, 0.21), *p* = 0.010*
Refreshed	*r* = 0.06 (−0.02,0.15), *p* = 0.168	*r* = 0.15 (0.07, 0.24), *p* < 0.001***	*r* = 0.15 (0.06, 0.23), *p* = 0.001**	*r* = 0.10 (0.01, 0.19), *p* = 0.031*
***Accessibility of NOEs at/near home***				
Total no. of NOEs can access from home	*r* = 0.282 (0.2, 0.36), *p* = < 0.001***	*r* = 0.083 (−0.01, 0.17) *p* = 0.065	*r* = 0.50 (0.43, 0.56), *p* < 0.001***	*r* = 0.49 (0.43, 0.56), *p* < 0.001***
Total no. of NOEs at home (e.g., *private or shared yard*)	*r* = 0.235 (0.15, 0.32), *p* = < 0.001***	*r* = 0.029 (−0.06, 0.12), *p* = 0.1	*r* = 0.41 (0.33, 0.48), *p* < 0.001***	*r* = 0.33 (0.25, 0.41), *p* < 0.001***
Total no. of NOEs near home (*beyond home within 10 min walk*)	*r* = 0.228 (0.14, 0.31), *p* = < 0.001***	*r* = 0.08 (−0.01, 0.17), *p* = 0.076	*r* = 0.41 (0.34, 0.48), *p* < 0.001***	*r* = 0.43 (0.36, 0.50), *p* < 0.001***
Access to 3 or more NOEs near home *(within 10 min walk)*	*r* = 0.199 (0.11, 0.28), *r* = < 0.001***	*r* = 0.124 (0.04, 0.21), *p* = 0.006**	*r* = 0.30 (0.22, 0.37), *p* < 0.001***	*r* = 0.33 (0.25, 0.41), *p* < 0.001***
***Total no. of barriers to NOE access from home***	*r* = −0.24 (−0.32, −0.15), *p* = < 0.001***	*r* = −0.15 (−0.23, −0.06), *p* = 0.001**	*r* = −0.10 (−0.19, −0.01), *p* = 0.023*	*r* = −0.05 (−0.13, 0.05) *p* = 0.36
No barriers	111/144 (77%)	127/141 (90%)		
1	127/207 (61%)	164/205 (80%)		
2	55/109 (50%)	81/108 (75%)		
3+	17/42 (40%)	29/40 (72%)		

*+Pearson correlation.*

p-values: ***p < 0.001; **p < 0.01; *p < 0.05.

When asked about the NOEs which were accessible to them both at and near their home (within a 10 min walk of home), respondents with higher quantity of NOEs for both at (*p* < 0.001; 95% CI: 0.15–0.32) and near home (*p* < 0.001, 95% CI: 0.14–0.31) were more likely to spend time outdoors on four or more days per week. Similarly, those with higher diversity of NOEs nearby (i.e., 3 or more different types of NOEs within a 10 min walk of home) were also significantly more likely to spend time outdoors on four or more days per week (*p* < 0.001, 95% CI: 0.11–0.28), but were also more likely to spend more than 30 min outdoors each outing (*p* = 0.006; 95% CI: 0.04–0.21). Access to and diversity of NOEs both at and near home were also highly correlated with use of NOEs both before and since the pandemic emerged (see Table 7).

The total number of barriers to accessing nature was also highly correlated with time outdoors and use of NOEs. The more barriers to accessing natural outdoor environments near home reported by respondents, the lower the time spent outdoors in both frequency (*p* < 0.001; 95% CI: −0.32 to −0.15) and duration (*p* = 0.001; 95% CI: −0.23 to −0.06). The number of perceived barriers to accessing natural environments from home also influenced use of NOEs before the pandemic (*p* = 0.023; 95% CI: −0.19 to −0.01) but not since (*p* = 0.36).

### Differences in Accessibility of Natural Outdoor Environments by Subgroups

The majority of respondents reported high access to NOEs at home via a private or shared yard (91%), or a private or shared deck, balcony or patio (80%) (see Table 8). For NOEs near home (within a 10-min walk), respondents could access an average of 2.92 (out of 7 options; SD = 1.69) different types of NOEs and 60% of all respondents reported nearby access to at least three different types of NOEs. Sixty percent or more reported easy access to public parks, nature trails, and rivers or streams.

**TABLE 8:** Differences in accessibility of NOEs at and near home by all participants and home setting and racial subgroups.

Types of NOEs	All	Home setting				Race		
		Rural	Suburb/Small town	Urban	p+	Non-White	White	p+
	*n* = 507 (%)	*n* = 121 (%)	*n* = 184 (%)	*n* = 143 (%)		*n* = 113 (%)	*n* = 394 (%)	
Private or shared yard	91	99	93	85	<0.001***	79	94	<0.001***
Deck, balcony, or patio	80	80	85	76	0.20	76	82	0.33
Public park, garden or orchard	59	35	57	83	<0.001***	65	57	0.33
Botanical garden, arboretum or nature center	15	7.4	18	15	0.056	22	13	0.08
Nature/Hiking trail	69	68	68	69	>0.9	68	70	0.87
Woodland or conservation area	49	61	46	38	<0.001***	45	50	0.52
River, stream, canal or waterfall	60	64	55	65	0.20	49	63	0.039
Lake, pond, or beach	36	48	32	29	0.008**	36	36	>0.9
Other	2	2.5	1.6	2.1	>0.9	3.5	1.5	0.33
**Total NOEs** (at or near home; 0–9 options) **mean (SD)**	**4.64 (1.84)**	**4.70 (1.79)**	**4.58 (1.82)**	**4.64 (1.70)**	**p++ = 0.9**	**4.44 (2.13)**	**4.69 (1.75)**	**p+++ = 0.33**
**Total NOEs at home** *(0–2 options)* **mean (SD)**	**1.71 (0.56)**	**1.79 (0.43)**	**1.79 (0.46)**	**1.61 (0.64)**	p++ = 0.008**	**1.55 (0.71)**	**1.76 (0.49)**	p+++ = < 0.001***
**Total NOEs near home** *(within 10 min walk; 0–7 options)* **mean (SD)**	**2.92 (1.69)**	**2.91 (1.70)**	**2.79 (1.78)**	**3.03 (1.48)**	**p++ = 0.52**	**2.89 (1.78)**	**2.93 (1.66)**	**p+++ = 0.87**
**3 or more NOEs available near home** *(within 10 min walk)*	**60%**	**59%**	**55%**	**65%**	**p++ = 0.29**	**57%**	**61%**	**p+++ = 0.52**

*+Fisher’s exact test across groups; adjusted p-values.*

*++ANOVA, adjusted p-values.*

*+++Paired t-test, adjusted p-values.*

p-values: ***p < 0.001; **p < 0.01; *p < 0.05.

There were significant differences in access to NOEs both at and near home by respondents living in different community settings. Fewer urban respondents reported having access to private or shared yards at home (*p* < 0.001) than respondents living in either rural or suburban/small town areas. Rural respondents reported significantly more access to NOEs such as woodlands (*p* < 0.001) and lakes, ponds or beaches (*p* = 0.008). Urban dwelling respondents reported significantly more access to public parks or gardens (*p* < 0.001) than suburban/small town and rural respondents.

The only significant difference in access to different types of NOEs by racial groups was the availability of outdoor spaces at home; non-White respondents reported significantly less access to private or shared yards than White respondents (*p* < 0.001), as well as water features such as rivers, streams, canals or waterfalls (*p* = 0.04). There were no significant differences between racial groups in the total number or diversity of NOEs near home.

When asked what features or conditions prevent or limit participants’ ability to spend time outdoors in nature when at home, including when working from home, respondents reported an average of 1.11 total barriers (SD = 0.97) (see Table 9). By far the most commonly cited barrier by all respondents was lack of time (51%), followed by having no one with whom to spend time outdoors (14%) and unfavorable environmental conditions (14%). A portion of respondents (7.7%) felt that NOEs near to their home felt unsafe or unwelcoming. When considering differences in barriers experienced across different respondent subgroups, there were no significant differences in the types of barriers experienced across different home settings. Urban dwelling respondents reported more total barriers on average than either rural or suburban/small town respondents, but these differences were not significant. Respondents with others living in their household reported a much higher average number of barriers to accessing natural environments (*p* < 0.001), and were more likely to report a lack of time (*p* < 0.001) as a barrier to spending time outdoors. Across racial subgroups, more non-White respondents reported that they do not have easy access to natural outdoor environments and that nearby NOEs feel unsafe or unwelcoming, but these differences were not significant. There was little differences in the total number of barriers reported across racial groups.

**TABLE 9:** Differences in barriers to accessing NOEs for all participants and home setting, living arrangement and racial subgroups.

What prevents or limits ability to spent time outdoors in nature during free time	All	Home setting				Living arrangements			Race		
		Rural	Suburb/Small town	Urban	p+	Live alone	Live with others	p+	Non-White	White	p+
	*n* = 507 (%)	*n* = 121 (%)	*n* = 184 (%)	*n* = 143 (%)		*n* = 24 (%)	*n* = 483 (%)		*n* = 113 (%)	*n* = 394 (%)	
Don’t have enough time	51	51	49	57	0.3	8.3	53	<0.001***	50	51	0.86
Don’t have easy access to nature	5.9	3.3	4.9	9.8	0.069	4.2	6	>0.9	11	4.6	0.15
Nearby NOEs feel unsafe or unwelcoming	7.7	5.8	6.5	11	0.2	4.2	7.9	>0.9	14	5.8	0.112
I have no one to go with; engage in outdoor activities with	14	12	15	17	0.4	0	15	0.14	18	13	0.56
Lack of daylight	6.7	8.3	9.2	4.2	0.2	4.2	6.8	>0.9	3.5	7.6	0.49
Workload	1	0	1.1	2.1	0.4	0	1	>0.9	0	1.3	0.82
Physical health issues	2.2	2.5	1.6	2.1	>0.9	0	2.3	>0.9	2.7	2	0.82
Mental health issues	1	0.8	1.1	1.4	>0.9	0	1	>0.9	0	1.3	0.82
Family responsibilities	3.6	5	3.8	3.5	0.9	0	3.7	>0.9	1.8	4.1	0.7
Prefer/Reliant on indoor/Digital activities	0.4	0	0.5	0	>0.9	0	0.4	>0.9	0.9	0.3	0.7
Unfavorable environmental conditions	14	13	16	16	0.8	0	15	0.14	8	16	0.15
Lack of motivation	1.4	1.7	0.5	2.8	0.3	0	1.4	>0.9	0	1.8	0.7
Other barriers	1.8	0	1.6	4.2	0.043	0	1.9	>0.9	0.9	2	0.82
**Mean total barriers mean (SD)**	**1.11 (0.97)**	**1.03 (0.87)**	**1.11 (0.91)**	**1.32 (1.09)**	**p++ = 0.28**	**0.21 (0.51)**	**1.15 (0.96)**	p+++ < 0.001***	**1.10 (1.00)**	**1.11 (0.96)**	**p+++ > 0.9**

*+Fisher’s exact test across groups; adjusted p-values.*

*++ANOVA, adjusted p-values.*

*+++Paired t-test, adjusted p-values.*

p-values: ***p < 0.001; **p < 0.01; * p < 0.05.

## Discussion

Employees in the United States experience high levels of stress due to their work and often feel ill equipped to manage these pressures (Liu, 2021). Work-related stress is of growing concern to employers because it has significant economic implications for organizations through employee dissatisfaction, reduced productivity, and lowered emotional and physical health (Kalia, 2002; Mirela and Madalina-Adriana, 2011). Millions of workdays are lost due to stress, anxiety, and depression-related illness (HSE, 2015).

During the changes and uncertainties presented by the COVID-19 pandemic, work expectations and environments shifted, as did non-work responsibilities as schools and daycares closed. Levels of stress increased, leaving many people at risk of short- and long-term stress-related illness. Staff members at institutions of higher learning report experiencing stress, anxiety and depression at even higher rates than the general United States workforce (Leal Filho et al., 2021).

Workplaces may choose to invest in practices that have been proven to reduce stress and anxiety. One well-researched stress management intervention is the use of natural outdoor environments (NOEs). As little as 10-min of time outdoors in nature has been shown to have a significant positive effect on psychological and physiological markers of mental well-being, including heart rate, blood pressure, salivary cortisol levels, mood, affect, happiness, stress, and attention (Meredith et al., 2019). Our study found that the majority of staff members spent more time outdoors in Spring 2020 when COVID first emerged than in Fall 2020, but that time outdoors in both periods in 2020 was greater for most than in Fall 2019, before the onset of the pandemic. Respondents reported spending time in NOEs for many reasons, including for exercise, to improve their physical health, and for enjoyment or recreation. However, the motivation which showed the most significant increase during COVID-19 was the self-identified use of NOEs for stress relief or improved mental health. The majority of respondents also identified that that they generally feel happier, less stressed, less anxious, and refreshed after spending time outdoors in nature. These results further reinforce the strong correlation between these positive feelings and higher reported levels of outdoor time and NOE usage and are consistent with other studies which demonstrated that contact with nature helped people cope with COVID-related challenges, especially for those under strict lockdown (Samuelsson et al., 2020; Venter et al., 2020; Pouso et al., 2021).

Compared to pre-COVID-times, many more respondents reported that their specific outdoor activities were of a restorative nature, such as resting, reading, meditating, and sleeping. These results suggest that many staff recognize both the restorative qualities and stress-relief benefits of time in nature, and that many intentionally used natural outdoor environments as a positive stress coping mechanisms during the pandemic. This finding is similar to a recent study showing English residents visited nature sites in unprecedented numbers during the COVID crisis, reportedly to help with their mental wellbeing and ability to cope (Robinson et al., 2021).

Because of concerns regarding the transmission of the virus from persons in close proximity, social outings in NOEs saw the greatest decline among staff respondents during the pandemic. These findings align with a study of nearly 5,000 Vermont residents early in the pandemic. They too showed increased participation in most outdoor activities except for camping and socializing with others, presumably in response to COVID restrictions (Morse et al., 2020). Analysis of changes in the levels of use or types of NOEs used by participants since the pandemic versus before its onset reveal that staff did not significantly increase or decrease their use of any particular NOE, but rather that many just used their habitual NOEs more frequently. This is in contrast to a study of residents of Freiberg, Germany, who noted that urban forests took on much greater importance for them during COVID-19, with many indicating that the forests had taken the place of public squares as social gathering sites (Weinbrenner et al., 2021).

Results, however, unequivocally illustrated that relative accessibility of NOEs significant impacted both the time staff spent outdoors and the number of NOEs used. The presence and diversity of outdoors spaces both at and near home were significant facilitators of the number of days staff spent outdoors in Fall 2020 and their overall use of NOEs. However, barriers to access were not limited to physical proximity. Other impediments included: social and environmental barriers, including lack of time, lack of companions to spend time with, unfavorable environmental conditions, and that local NOEs felt unsafe. These findings provide important insights that can inform targeted intervention strategies which both educational institutions and community planners can utilize to increase the ease and comfort with which employees and citizens can spend restorative time outdoors in nature.

Study findings also reinforce that lack of and barriers to access to outdoor green spaces are not experienced equally across staff respondents. Urban staff members had less access to outdoor spaces such as home yards or decks/patios yet greater access to public parks and gardens than staff living in rural or suburban/small town areas, reflecting the differences in land use planning, density and pedestrian networks available in differing community settings. Urban staff also faced more overall barriers to accessing NOEs than their colleagues. These findings emphasize that the home community setting where staff living can significantly impact the quantity and types of NOEs to which they have access, as well as the barriers to spending regular time outdoors, particularly for those who may continue to work from home. Increased break time and flexibility during the workday could help to minimize barriers to spending time in NOEs for both home and campus-based staff. For staff who have or are transitioning back to campus-based work, some of these gaps could be minimized by increasing opportunities for staff to spend time outdoors in nature during their workday on campus.

Additionally, non-White staff reported having less access to NOEs at home than did White staff, and generally spent less time in NOEs than did their White colleagues. This may have been due to various barriers non-White study participants experience or perceive, such as living farther from NOEs, lacking convenient transportation to travel to such sites, or feeling less safe or unwelcome when in parks or woodlands. This finding adds to well-documented disparities regarding green access for Black, Indigenous and People of Color (BIPOC) versus White individuals (Hong and Anderson, 2006; Byrne, 2012; Rigolon and Németh, 2018; Borunda, 2020).

The great majority (95%) of respondents were living with others in their household during the pandemic, and they reported facing greater overall barriers to use of NOEs than did those living alone, particularly noting lack of enough time, which may be related to home responsibilities associated with dependents. Identifying subgroups of employees who have less access to NOEs, or who face more barriers to spending time outdoors, can also help guide directed efforts to encourage and support time outdoors. Large-scale employers such as universities can also minimize these disparities by providing easy access to diverse, safe outdoors spaces on the property and encouraging regular time outdoors during the workday via shifts in institutional culture and policies.

Generally, individual staff perceptions about their outdoor experiences strongly correlated to the frequency and duration of those experiences. Those that identified higher positive feelings after time outdoors spent more days and more time outdoors during each outing, and also used a greater diversity of NOEs. Conversely, the more barriers to accessing nature individuals noted, the less time in frequency and duration they spent outdoors and the fewer NOEs they used. Since spending time in nature, unlike having adequate food and shelter, is not an essential human need, any physical or perceptual barriers can inhibit individuals’ use of NOEs and the benefits they provide.

Encouragingly, results from this study indicate that a large proportion of staff surveyed are proactively spending time in natural outdoor environments to support their health, in particular their mental well-being. With a large number of staff noting relatively easy access to nature and intentionally choosing to spend time outdoors to improve their mental health, spending time outdoors likely served as a protective factor for many staff against some of the potentially detrimental effects of the pandemic. COVID-related restrictions which made indoor gathering difficult may also have raised awareness among staff about the benefits of nature as well as the natural resources available to them nearby. However, a significant portion of staff noted they had difficulties accessing or spending time in nature, and this was particularly true for those living with dependents and others in their household, and BIPOC staff.

### Limitations and Future Research

The authors recognize a number of limitations related to this study. First, the number of survey respondents (507) represents a relatively small sample from a single institution. This might limit external generalizability. Second, the sample represents a small proportion of all invited to respond (7% of people on the newsletter distribution list). The sample represents a higher-than expected proportion of female respondents, when compared to the overall staff population. Furthermore, individuals who responded to the survey self-selected to participate, and may be biased toward physical activity and time outdoors, as the survey invitation was distributed via a wellness newsletter. Also to note is that while the proportion of non-White staff respondents was higher than the total percentage of non-White staff at this university, we should be cautious of generalizing actions and attitudes of non-White staff members based on the small sample, particularly as analysis required the clustering of all non-White staff members, which in turn may cloak differences in behaviors and perceptions across different racial or ethnic groups. These issues may therefore reduce the applicability of findings to the full staff community. Finally, the survey looked to measure change, but was only administered at one time point (November–December 2020), and respondents were asked to compare current actions and activities to those months (April–July 2020) and a year (Fall 2020) ago. Recall bias might have influenced the results.

It is also important to note that while we were interested in the use of time in nature to manage stress and mental health, we intentionally avoided asking direct questions to staff in the survey about their mental health. We judged that the greater sensitivity and invasiveness of such questions would lead to higher levels of discomfort and lack of privacy and would like result in lower response rates. This strategy precluded the analysis from directly correlating time outdoors with improved mental health. However, responses to several questions related to motivations for spending time in NOEs, and how staff felt after time outdoors, suggest that this would likely be the case. Future work could attempt to solicit these data in order to tie these outcomes more explicitly together.

The strong relationships between motivations for and feelings after spending in nature, and time spent outdoors in natural outdoor environments, suggests that facilitating time outdoors in nature among staff may result in both healthier workforces and workplaces. In a post-COVID era, we recommend that future research focus on the use of NOEs by staff at large institutions such as universities during regular work hours to examine impacts on staff health and performance. For example, do institutions with greater numbers of and more diverse NOEs see lower rates of job dissatisfaction, psychological problems, employee stress and burnout? Can the scheduling of employee breaks in green settings increase productivity or feelings of loyalty to the institution? Do staff members who increase their time in NOEs during the workday display changes to their overall environmental attitudes? Future research could also dig more deeply into the complexities of staff use and perceptions of nature and outdoor environments, and further unpack the diverse and nuanced barriers which can limit employees’ abilities to leverage time outdoors in nature as a coping and stress reduction strategy.

### Concluding Remarks

Based on this study, staff members did seek out and utilize NOEs during COVID-related lockdowns with greater frequency than before the pandemic. While the motivations varied by individual, overall respondents were more likely to seek out such sites for stress relief or recreation, and less for social contact or group gatherings. Improving access to green spaces on campus by providing flexibility with break time, increased promotion of their availability, or reduction of physical barriers, can benefit the well-being of staff members.

This study emphasizes that in examining the ability of employees to spend time in natural outdoor environments, we must also consider their unique social and environmental contexts. Factors including the type of community they live in, the people they live with, their work location, their motivations and their race can all impact the particular facilitators or barriers to experiencing natural outdoor environments.

While some university staff continue to work fully or partially from home, many are returning to campus-based work. Universities and other large-scale employers are therefore faced with a prime opportunity to not only improve the overall well-being of the staff, but to reduce disparities in well-being and access to nature among their employees. By working to both increase the availability of safe and supportive natural outdoor environments available on site, as well as identifying and working to overcome physical, social and environmental barriers to spending time outdoors faced by employees, universities and other employers could help to foster healthy, productive and more resilient workers and work environments during and beyond the COVID-19 pandemic.

## Data Availability Statement

The raw data supporting the conclusions of this article will be made available by the authors, without undue reservation.

## Ethics Statement

The studies involving human participants were reviewed and approved by the Cornell University Institutional Review Board. The patients/participants provided their written informed consent to participate in this study.

## Author Contributions

All authors contributed to original draft preparation, conceptualization and development of the study protocol and survey tool, data review and interpretation, and reviewing and editing.

## Conflict of Interest

The authors declare that the research was conducted in the absence of any commercial or financial relationships that could be construed as a potential conflict of interest.

## Publisher’s Note

All claims expressed in this article are solely those of the authors and do not necessarily represent those of their affiliated organizations, or those of the publisher, the editors and the reviewers. Any product that may be evaluated in this article, or claim that may be made by its manufacturer, is not guaranteed or endorsed by the publisher.
