# Supplementary material for: Time Outdoors in Nature to Improve Staff Well-Being: Examining Changes in Behaviors and Motivations Among University Staff in the Use of Natural Outdoor Environments Since the Emergence of the COVID-19 Pandemic
Source: Front Psychol. 2022 Jul 22;13:869122. doi: 10.3389/fpsyg.2022.869122 (PMC9354818; doi:10.3389/fpsyg.2022.869122)
Supplement: Supplementary file 1 [file Data_Sheet_1.PDF]

Staff Time Outdoors in Nature

Greetings! We invite you to participate in a short survey to tell us about your time in nature or natural outdoor environments both before and during the COVID-19 pandemic. Responses from this survey will help inform efforts to keep xx staff healthy and well during the 2020-21 academic year and beyond.   This survey should take 10-15 minutes or less to complete. Your responses to these questions are anonymous. No identifiable data will be recorded, and data will only be presented in summary reports.   Participation in this survey is voluntary, and you can skip any questions that you prefer not to answer.   As a thank you for your participation, at the end of the survey you'll be given the opportunity to enter your xx email address (not linked to this survey) in a draw for $50 grocery gift cards. Entering into the draw is optional and confidential.

To view a fuller outline of the study, along with its risks and benefits, click on this link: Full Letter of Information

---

If you agree to participate in this survey, please select 'Yes'

○ Yes, Let's Go! (1)

○ No thank you! (2)

Skip To: End of Survey If If you agree to participate in this survey, please select 'Yes'   = No thank you!

TIME IN & ACCESS TO NATURE

For the purposes of this survey, time spent 'outdoors in nature' refers to any time you spent outdoors specifically where you have some contact with or experience of nature.   This includes time spent in: backyards, gardens, parks, green spaces, trails, woodlands, rivers or canals, lakes, beaches, nature/conservation areas, etc.

Q Tell us whether you have access to EACH of these natural outdoor environments at or near your home (within a 10 min walk):

- ☐ Private or shared yard (1)
- ☐ Deck, balcony or patio (2)
- ☐ Public park, garden or orchard (5)
- ☐ Botanical garden, arboretum or nature center (7)
- ☐ Nature / hiking trail (8)
- ☐ Woodland or conservation area (9)
- ☐ River, stream, canal or waterfall (10)
- ☐ Lake, pond or beach (11)
- ☐ Other (please specify): (13) ____
- ☐ None of the above (14)

Q For EACH of these natural outdoor environments, please tell us whether or not you used them during EACH of the noted time periods: (check ALL that apply for EACH column)

	Natural / Outdoor Environments	
	Typically used or visited BEFORE COVID-19 emerged (1)	Used or visited SINCE COVID-19 emerged (March 2020 - present) (2)

Private or shared yard (1)	☐	☐
Deck, balcony or patio (2)	☐	☐
Public park, garden or orchard (5)	☐	☐
Botanical garden, arboretum or nature center (7)	☐	☐
Nature / hiking trail (8)	☐	☐
Woodland or conservation area (9)	☐	☐
River, stream, canal or waterfall (10)	☐	☐
Lake, pond or beach (11)	☐	☐
Other (please specify): (13)	☐	☐
None of the above (14)	☐	☐

Q Over the last 2 months (Fall 2020), how many days per week have you typically spent some time outdoors (where you have had some contact with nature):

- ○ No days (8)
- ○ 1 day (9)
- ○ 2-3 days (10)
- ○ 4-6 days (11)
- ○ 7 days (12)

Skip To: Q2.7 If Over the last 2 months (Fall 2020), how many days per week have you typically spent some time out... = No days

---

Q On days you spent time outdoors during these last 2 months, about how much total time per day would you typically spend outdoors?

- ○ Less than 15 min (6)
- ○ 15-30 min (7)
- ○ 31min - 1hr (8)
- ○ More than 1 hr (9)

Q Over the past 2 months (Fall 2020), compared to weekdays, how much time per day would you typically spend outdoors in nature on weekend days?

- ○ Less time (8)
- ○ About the same time (9)
- ○ More time (10)
- ○ I don't spend time in nature/outdoors on the weekend (11)

Q Thinking back to the months just after COVID-19 emerged (April-July 2020) and much of campus was closed, how much time did you spend outdoors in nature compared to the past 2 months (Fall 2020)?

- ◯ Less time (1)
- ◯ About the same time (2)
- ◯ More time (3)
- ◯ I don't know (4)

Display This Question:

If Thinking back to the months just after COVID-19 emerged (April-July 2020) and much of campus was... = Less time

Or Thinking back to the months just after COVID-19 emerged (April-July 2020) and much of campus was... = More time

Q What influenced this change?

____

Q Thinking back to a year ago (Fall 2019; pre-COVID), how much time did you spend outdoors in nature compared to Fall 2020?

- ◯ Less time (1)
- ◯ About the same time (2)
- ◯ More time (3)
- ◯ I don't know (4)

Display This Question:

If Thinking back to a year ago (Fall 2019; pre-COVID), how much time did you spend outdoors in natur... = Less time

Or Thinking back to a year ago (Fall 2019; pre-COVID), how much time did you spend outdoors in natur... = More time

Q What influenced this change?

____

ACTIVITIES OUTDOORS IN NATURE

Q What activities did you typically do outdoors in nature? (check ALL that apply for each time period):

	Before COVID-19 (1)	During COVID-19 (2)
Social activities: like gatherings or dining with others (2)	▢	▢
Lower intensity activities: like walking, hiking, gardening, birdwatching, fishing (3)	▢	▢
Higher intensity activities: like running, biking, rock-climbing, kayaking (4)	▢	▢
Restorative activities: like resting, reading, meditating, sleeping (1)	▢	▢
Other (please specify any other activities): (9)	▢	▢
I didn't spend any time in nature/outdoors (10)	▢	▢

Q Who did you spend time with outdoors in nature? (select ALL that apply for each time period):

	Before COVID-19 (1)	During COVID-19 (2)
Myself alone (1)	☐	☐
My family (2)	☐	☐
My friends / neighbors (5)	☐	☐
My colleagues / workmates (7)	☐	☐
Others (please specify): (8)	☐	☐

BENEFITS & BARRIERS

Q Why did you spend time in outdoors in nature? (select ALL that apply for each time period):

	During COVID-19 (1)	Before COVID-19 (2)
For exercise / improved physical health (1)	☐	☐
For stress relief / improved mental health (2)	☐	☐
For fun / recreation (3)	☐	☐
To have contact with nature (6)	☐	☐
To spend time with family (4)	☐	☐
To socialize with friends (5)	☐	☐
Other (please specify): (7)	☐	☐

Q How much do you agree with each statement. After spending time outdoors in nature I feel:

	Strongly agree (1)	Somewhat agree (2)	Neither agree nor disagree (3)	Somewhat disagree (4)	Strongly disagree (5)	I don't know (6)
Healthier (1)	o	o	o	o	o	o
Happier (2)	o	o	o	o	o	o
Less stressed / anxious (3)	o	o	o	o	o	o
More focused (4)	o	o	o	o	o	o
Refreshed (5)	o	o	o	o	o	o

Q How do you generally feel about nature?

	Definitely (1)	Somewhat (2)	Not at all (3)	I don't know (4)
I am drawn to nature (1)	o	o	o	o
I consciously try to spend time in nature (2)	o	o	o	o
Spending time in nature is essential to my well-being (5)	o	o	o	o

Q When at home (including when working from home), does anything prevent or limit your ability to spend time outdoors in nature during your free time? (check ALL that apply):

- ☐ I don't have enough time (3)
- ☐ I don't have easy access to nature (4)
- ☐ Nearby outdoor/natural spaces feel unsafe or unwelcoming (7)
- ☐ I have no one to go with; engage in outdoor activities with (8)
- ☐ Other barriers (please specify): (9) ____
- ☐ I am not interested in spending time outdoors in nature (1)
- ☐ Nothing / little prevents or limits me (10)

Q **WORK ENVIRONMENT**

Q When working on campus pre-COVID, how many days per week would you typically spend some time outdoors in nature on breaks during your workday?

- ○ 0 days (1)
- ○ 1 day (2)
- ○ 2-3 days (3)
- ○ 4-5 days (4)

Q Since COVID-19 emerged, where have you been working?

- ◯ I have been working from home the whole time; I have not been on campus (1)
- ◯ I worked primarily at home with occasional visits to campus (2)
- ◯ I split my time between home and campus (3)
- ◯ I worked primarily at home after March 2020, but returned to full campus-based work in Fall 2020 (4)
- ◯ I continued to work primarily on campus (5)
- ◯ Other (please specify): (6) ____

Display This Question:

If Since COVID-19 emerged, where have you been working? != I continued to work primarily on campus

Q When working from home, do you spend more or less time outdoors in nature during your workday than when you work from campus?

- ◯ Less time (1)
- ◯ About the same (2)
- ◯ More time (3)
- ◯ I don't know (4)

Display This Question:

If Since COVID-19 emerged, where have you been working? != I have been working from home the whole time; I have not been on campus

Q Do you spend more or less time outdoors in nature when working on campus now than pre-COVID?

- ◯ Less time (1)
- ◯ About the same time (2)
- ◯ More time (3)
- ◯ I don't know (4)

Q What do you typically do outdoors in nature on campus? (check ALL that apply):

- ☐ eat lunch / take coffee break outdoors (1)
- ☐ take a walk, stroll or hike (2)
- ☐ gather or meet with friends / workmates (3)
- ☐ rest / relax / read (4)
- ☐ other (please specify): (5) ____
- ☐ I don't typically spend time outdoors in nature on campus (6)

Q During your workday on campus, does anything prevent or limit your ability to spend time outdoors in nature? (check ALL that apply):

- ☐ I am not interested in spending time outdoors in nature (1)
- ☐ I don't have enough time (2)
- ☐ I am discouraged from leaving my building during breaks (3)
- ☐ There are no natural/green spaces nearby / easily accessible (4)
- ☐ Nearby natural/green spaces are unappealing or of low quality (9)
- ☐ Not enough protection from weather (like sun, wind, rain, etc) (10)
- ☐ Other barriers (please specify): (11) ____
- ☐ Nothing / Little prevents or limits me (12)

Q Would you like to increase the frequency or time you spend outdoors in nature when you are working from campus (now or in the future)?

- ○ Definitely (1)
- ○ Somewhat (2)
- ○ Not at all (3)
- ○ I don't know (4)

Display This Question:

If Would you like to increase the frequency or time you spend outdoors in nature when you are workin... != Not at all

Q What would make that easier or more enjoyable?

____

Q Are there any outdoor or nature-based activities or habits that you've developed since COVID-19 emerged that you would like to continue (such as going for a nature walk on your lunch; taking the family to a park or trail on the weekends; more regular gardening)?

- ○ Yes (what new activities) (1) ____
- ○ No (2)
- ○ I am not sure (3)

Q6.1 **NATURE-BASED FAMILY ACTIVITIES**

Q Which other individuals are currently living <u>in your household</u>? (check ALL that apply)

- ☐ I currently live by myself (10)
- ☐ Children – daycare/pre-school age (1)
- ☐ Children – elementary school age (2)
- ☐ Youth – middle school age (3)
- ☐ Youth – high school age (4)
- ☐ Partner/spouse (5)
- ☐ Parent(s) or older family member (60+ yrs) (6)
- ☐ Other family members (7)
- ☐ Roommate / Friend (8)
- ☐ Other (please specify) (9) ____

Display This Question:

If Which other individuals are currently living in your household? (check ALL that apply) != I currently live by myself

Or Which other individuals are currently living in your household? (check ALL that apply) != Roommate / Friend

Q Before COVID-19, how often would you engage in family leisure activities outdoors in nature (such as gardening together in the backyard; going for walk/hike in nature; visiting a park or playground)?

- Very frequently (1)
- Frequently (2)
- Occasionally (3)
- Rarely (7)
- Never (8)
- I don't know (4)

Display This Question:

If Before COVID-19, how often would you engage in family leisure activities outdoors in nature (such... != Never

Q What kind of leisure activities outdoors in nature do you fairly regularly do with your family (before OR during COVID-19)? (Check ALL that apply):

- ▢ We don’t typically spend family leisure time outdoors in nature (1)
- ▢ Walking, hiking (2)
- ▢ Running, biking, rockclimbing (3)
- ▢ Kayaking, canoeing, boating, SUP, swimming (5)
- ▢ Gardening, birdwatching, fruit-picking (6)
- ▢ Relaxing, reading, picnicking (7)
- ▢ Camping (9)
- ▢ Fishing, hunting (10)
- ▢ Other family rituals / traditions outdoors in nature: (11) ____

Skip To: End of Block If What kind of leisure activities outdoors in nature do you fairly regularly do with your family (b... = We don’t typically spend family leisure time outdoors in nature

Q Thinking about your family / household: Spending time together outdoors in nature ...

	Strongly agree (1)	Somewhat agree (2)	Neither agree nor disagree (3)	Somewhat disagree (4)	Strongly disagree (5)	N/A or I don't know (6)
is important for the well-being of my children (1)	o	o	o	o	o	o
is important for the well-being of older adults in my household (2)	o	o	o	o	o	o
has positive benefits for us as a family (3)	o	o	o	o	o	o
has increased since COVID-19 has emerged (4)	o	o	o	o	o	o
has been easier since COVID-19 emerged (5)	o	o	o	o	o	o

DEMOGRAPHICS

Q How do you identify?

- ○ Male (1)
- ○ Female (2)
- ○ Other (3)
- ○ Prefer not to respond (4)

Q How long have you worked at [X University]?

- ○ Less than 2 years (1)
- ○ More than 2 but less than 5 years (2)
- ○ More than 5 but less than 10 years (3)
- ○ 10 or more years (4)

Q Please indicate your current [X University] employment status (check <u>ALL</u> that apply)

- ☐ Full-time (2)
- ☐ Part-time (3)
- ☐ Permanent (4)
- ☐ Contract (5)
- ☐ Temporary / Casual (6)
- ☐ Seasonal (7)
- ☐ Other (please specify): (1)
____

Q How is your position categorized?

- ○ Hourly (Non-Exempt) (1)
- ○ Salaried (Exempt) (2)
- ○ Other (please specify): (3) ____

Q When working <u>from campus</u>, how often do you <u>work outdoors</u> as part of your position responsibilities?

- ○ Most of the time (1)
- ○ Some of the time (2)
- ○ Never or rarely (3)

Q Which option best describes where you currently live?

- Larger city or urban center
- Smaller city or urban center
- Suburb or just outside an urban center
- Small town or village
- Rural area

Q With which ethnicities or races do you identify (Check ALL that apply):

- Black or African American (1)
- Hispanic or Latina/o (2)
- American Indian or Alaska Native (3)
- Asian (4)
- Native Hawaiian and Pacific Islander (5)
- White (6)
- Other (please specify): (7) ________________
- Prefer not to answer (8)
